# FTSPlot: Fast Time Series Visualization for Large Datasets

**DOI:** 10.1371/journal.pone.0094694

**Published:** 2014-04-14

**Authors:** Michael Riss

**Affiliations:** Department of ETSEIB, Technical University of Catalonia (UPC), Barcelona, Spain; Leuven University, Belgium

## Abstract

The analysis of electrophysiological recordings often involves visual inspection of time series data to locate specific experiment epochs, mask artifacts, and verify the results of signal processing steps, such as filtering or spike detection. Long-term experiments with continuous data acquisition generate large amounts of data. Rapid browsing through these massive datasets poses a challenge to conventional data plotting software because the plotting time increases proportionately to the increase in the volume of data. This paper presents FTSPlot, which is a visualization concept for large-scale time series datasets using techniques from the field of high performance computer graphics, such as hierarchic level of detail and out-of-core data handling. In a preprocessing step, time series data, event, and interval annotations are converted into an optimized data format, which then permits fast, interactive visualization. The preprocessing step has a computational complexity of 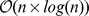; the visualization itself can be done with a complexity of 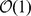 and is therefore independent of the amount of data. A demonstration prototype has been implemented and benchmarks show that the technology is capable of displaying large amounts of time series data, event, and interval annotations lag-free with 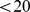 ms. The current 64-bit implementation theoretically supports datasets with up to 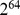 bytes, on the x86_64 architecture currently up to 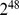 bytes are supported, and benchmarks have been conducted with 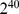 bytes/1 TiB or 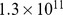 double precision samples. The presented software is freely available and can be included as a Qt GUI component in future software projects, providing a standard visualization method for long-term electrophysiological experiments.

## Introduction

For understanding long-term neuronal processes, such as growth, plasticity/learning, degeneration and regeneration, it is necessary to monitor neuron activity over long periods of time. Currently, numerous single and multichannel extra-cellular electrophysiology techniques offer long-term recording capability.

For in vivo experiments, protruding electrode arrays are available to record extra-cellular potentials from cortical areas for over two years [1]–[4], microwire bundles can be used to record extra-cellular potentials from deeper areas within the brain for more than one year [5]–[8], and cuff/cone electrodes provide recordings from enclosed nerves and neural tissue with stable neuron-electrode connection for up to three years [9]–[11].

In vitro culture dishes with integrated multi-electrode arrays (MEAs) are used for experiments on neuron cultures [12]–[14] and can keep the cultures alive for 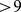 months [15]. The cultures can be patterned either chemically [16] or physically [17]–[20], enabling circuit level observation of neuronal development and plasticity.

However, continuous long-term recording results in huge datasets that pose a challenge to storage and analysis of the data. For example, when recording at 10 kHz and with 8 bytes per sample, each recording channel generates 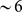 gibibyte (GiB, IEC nomenclature, [21]) per day. In the past, high costs of data storage were prohibitive for saving the complete recording in raw data format. Algorithms were developed to conduct filtering, spike detection, and classification during the experiment in real-time, and only short sweeps, time stamps, and selected parameters of the classified spikes were saved to the hard disk [22], [23].

Nowadays, the modern off-the-shelf high-capacity hard drives have made raw data storage of long-term recordings feasible at a relatively low cost. Raw data storage offers the advantage that the data can be analyzed after the experiment with access to the complete recording. This permits the iterative refinement of the analysis process, testing of alternative analysis algorithms, identification and processing of unexpected artifacts, and the study of neurons displaying time-varying activity patterns.

During iterative data analysis, the visual inspection of the time series data is a reoccurring work step. Initially, artifacts and/or noise need to be identified and excluded from further analysis, for example, incomplete power line shielding or manipulation of the experiment setup. Subsequently, results of the processing steps, such as filtering, spike detection, and spike sorting need to be verified. Owing to the interactive nature of such analysis, the user should be able to quickly navigate through the data and view it at different magnification levels.

This requirement poses a challenge to traditional plotting programs. Although there are various ways to visualize the time series data [24], the most commonly used method for electrophysiological data is the line plot. The canonical approach for a line plot is to project the samples one by one onto the canvas and connect the resulting points with lines. Therefore, the plotting time depends on the amount of data: more data results in longer plotting time; the canonical algorithm has linear complexity; in O-notation [25], [26] expressed as 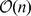. Owing to the large amounts of data produced by long-term recordings, the canonical plotting method cannot offer the necessary performance despite the impressive computational power of the current processors and graphic cards; therefore, a different algorithmic approach is required.

Currently, there are several techniques that address the challenge of displaying large time series datasets on finite-resolution displays. A movable virtual “lens” can be used to simultaneously display the data both at the overview and detailed levels. A section of the dataset is magnified and displayed in great detail within the overview, dynamically pushing aside the rest of the data and thereby creating a “lens effect.” Handling of datasets with up to 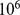 data points has been reported with this method [27], [28].

Binning can be used to display time series data at multiple levels of aggregation. The data is accumulated in a predefined number of bins and various parameters are calculated for each bin, for example, mean value, minimum/maximum value, and standard deviation. These values are then plotted instead of the original, complete dataset, thereby permitting, depending on the number of bins, visualization of the data at different aggregation levels. This method has been shown to handle 50,000 data points [29].

In the field of 3D computer graphics, similar problems with displaying large data amounts have been solved in the past by combining various strategies.

The level-of-detail (LoD) methods are based on the fact that complex 3D models often have more detail than the monitor can display with its fixed resolution and pixel size [30], [31]. This especially applies to scenes in which the object is far away and occupies only a small area on the monitor. The LoD method generates for each original, high-detail 3D model several additional 3D models with successively reduced geometric complexity. Depending on the size occupied by the object on the monitor, the algorithm selects the 3D model that still provides the maximum amount of detail that the monitor can display but avoids excess detail levels that go beyond the capabilities of the monitor.

This idea is further refined in surface splatting methods [32], [33]. These methods are used for models that store information about the object surface as a high density point cloud without connectivity. This permits the polygon representation to be abandoned and only the point cloud is projected as elliptic “splats” onto the pixel matrix of the monitor.

Handling the huge amount of data is an additional challenge. The size of high detail models usually exceeds the memory capacity of video cards and the main memory; therefore, the bulk model data has to remain on the hard disk. However, optimal performance can only be achieved by processing the data in the fast access memory levels (main and graphic card memory, and caches) [34]. Therefore, it is necessary to load the used data segments on demand into the main and graphic card memory as the user navigates through the model [35], [36].

The FTSPlot project shows how such methods can be applied and extended to achieve fast, interactive display of large-scale electrophysiological time series data, event, and interval annotations.

## Materials and Methods

### Overview

**Figure 1:**
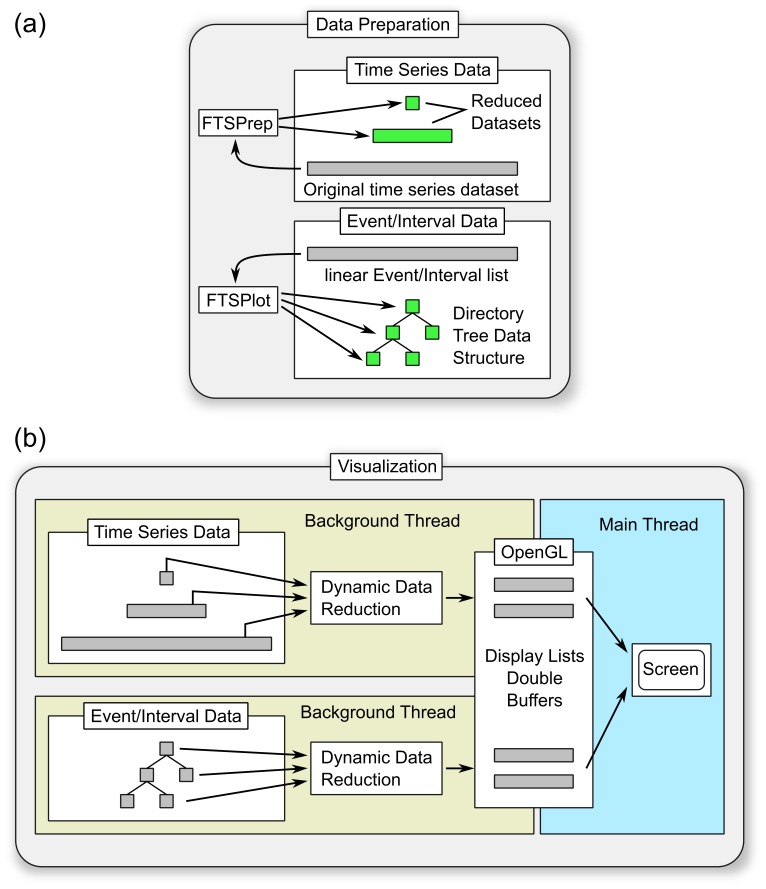
FTSPlot overview. (a) Data is prepared in an offline processing step. Time series data is reduced with the batch tool FTSPrep in several steps to form a static data reduction pyramid. Linear event/interval lists get converted to a hierarchical directory tree data structure directly in the FTSPlot program. (b) During visualization each dataset gets read by a separate background thread from the hard disk; further dynamic data reduction is applied to match the current zoom level of the visualization; the resulting data section is stored in the graphics system as an OpenGL display list. The main thread uses the prepared display lists to quickly draw the data to the screen. OpenGL display list double buffering allows simultaneous painting of the current display lists by the main thread while new display lists get prepared by the background threads.

Fig. 1 shows an overview of the FTSPlot concept. The original datasets are preprocessed to create static hierarchic data representations that are stored on the hard drive.

During visualization, the main thread receives user input (panning/zooming) and quickly paints the current data section, which is stored in a small OpenGL display list. Every time the user navigates towards the limits of the current display list, the main thread requests a new data section at the required detail level (zoom level) from the background thread. The background thread reads the data from the adequate static data reduction level, applies further data reduction (dynamic data reduction) to exactly match the needed detail level, and stores the new data section in the second OpenGL display list of the graphics subsystem. Once completed, the main thread switches the two display lists and can now present the new data section to the user.

### Time series data

FTSPlot works with electrophysiological datasets that store a single recording channel as a series of floating point values (IEEE 754, 64 bit).

#### Basic data reduction step

**Figure 2:**
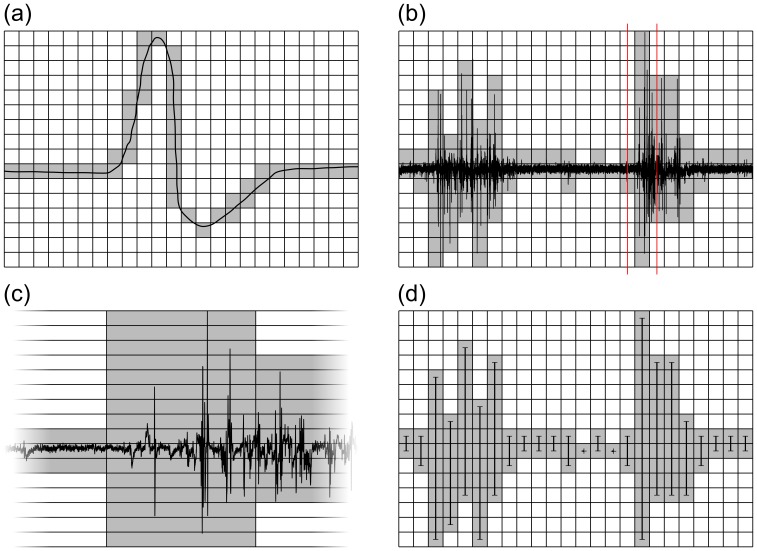
Time series data reduction. The basic principle of data reduction to accelerate time series plotting: (a) A computer display uses discrete pixels to display a graph. (b) Plotting large amounts of data results in overdraw; several data points get plotted onto the same pixel column. A zoomed version of the marked area (red lines) in (b) is shown in (c) as an example for the amount of detail that can collapse onto a single pixel column. (d) Replacing the original time series dataset with a reduced dataset consisting of minimum and maximum values for each pixel column gives the same visual result. The reduced dataset in this example consists of only 48 data points compared to the original time series with 38,866 samples in (b) and can therefore be plotted considerably faster.

The key technique underlying most hierarchical visualization techniques is the reduction of detail in the scene/model to accelerate plotting but without compromising the final visual result. This is possible because a computer display discretizes the image into pixels (Fig. 2 (a)). When a large amount of data is being plotted onto a computer display, the finite resolution of the display imposes a limit to the amount of details that can be displayed. If there are more data points than pixels, several data points get projected onto the same pixel (Fig. 2 (b)). This permits the use of a replacement representation that has less detail but yields the same visual result; this is often also called proxy representation/model [37], [38]. For FTSPlot, vertical lines have been chosen as the proxy representation for time series data (Fig. 2 (c)). Each line represents an interval of the time series dataset. The minimum and maximum values in the interval define the vertical positions of the two ends of the vertical line, and the average time-value defines the horizontal position. As long as the intervals are smaller than the pixel columns on the monitor, the visual result is indistinguishable from the normal, canonical visualization method.

This basic data reduction by proxy representation is the central algorithmic technique used in FTSPlot to accelerate data visualization. Instead of depending on the amount of data, the plotting time now depends on the resolution of the computer display. Therefore, because the display resolution is constant, the algorithm also has constant complexity—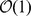.

Before visualization, however, the reduced dataset needs to be computed, which is a task of linear complexity—
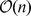
—as all the data points need to be processed to find the minimum and maximum values of the intervals. This step needs to be performed only once and it can be run unsupervised as a batch process before starting the visualization.

#### Data reduction hierarchy

The basic data reduction step described in the previous section solves the problem of quickly displaying time series data at one predefined magnification level. Scrolling panning is possible; however, dynamic zooming is not. When zooming into the data, the vertical lines would eventually become visible, revealing the data reduction trick and destroying the impression of looking at proper time series data. When zooming out, more and more vertical lines would get projected onto single pixel columns, which would linearly increase the plotting time and once again result in a linear complexity—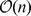.

**Figure 3:**
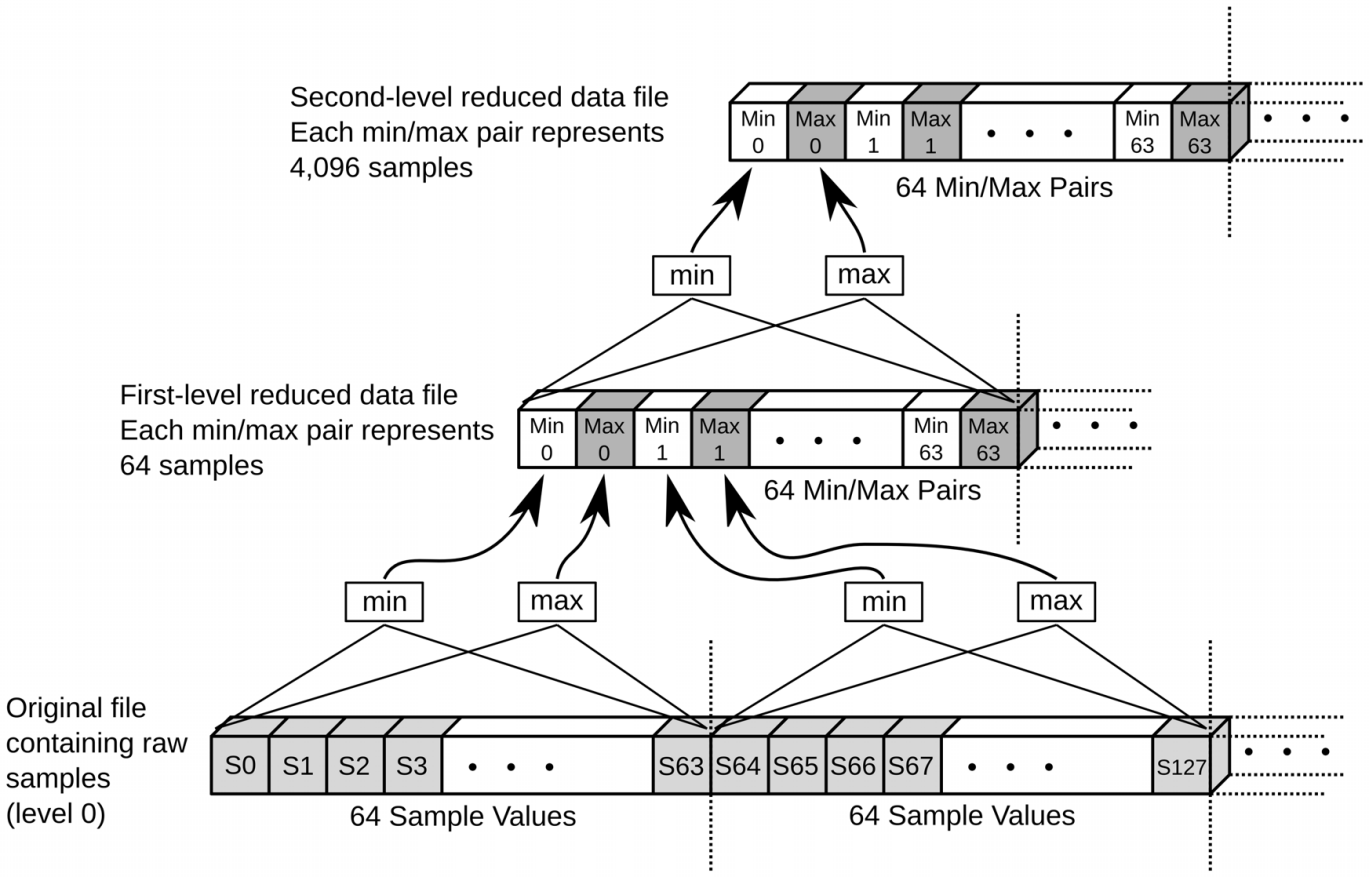
Time series data reduction pyramid. The data reduction pyramid for time series datasets and a THINNING FACTOR of 64. The original file with the samples is shown at the bottom. For each block of 64 samples, the minimum and maximum values get computed and stored in the file with the first-level reduced dataset (middle). The computation of the second-level reduced dataset is similar; for each block of 64 min/max pairs on the first level, the minimum and maximum value is computed and stored on the second level.

To solve this problem, the data gets reduced in several steps to create a static data reduction pyramid (Fig. 3) on the hard disk. The reduction factors of the pyramid levels are defined by 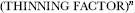, with the THINNING FACTOR being a user configurable parameter (needs to be a power of 2) and 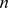 being the pyramid level starting with 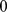 for the level of the original dataset.

#### Time series display list generation

During visualization, the background thread (Fig. 1 (b)) receives requests to prepare data sections for the main thread at specific reduction factors (depending on the current zoom level of the visualization). It chooses the closest static data reduction level in the pyramid “below” the requested reduction factor, that is, the closest static data reduction level that still contains more detail than requested. With the dynamic data reduction step, the data then gets further reduced by an additional reduction factor to match the reduction factor needed for visualization.

Example: Assuming a THINNING FACTOR of 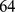, the first static reduction factor is 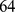, the second 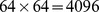, etc. If during visualization, a reduction factor of 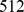 is requested, the algorithm would choose the first static reduction factor (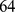) and then dynamically reduce the data with an additional reduction factor of 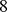 (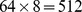).

The computational cost for the dynamic data reduction depends on the additional reduction factor; the bigger the additional reduction factor, the more the data needs to be searched to find the minimum and maximum values. However, the additional reduction factor cannot become bigger than the THINNING FACTOR (the distance between two adjacent static reduction factors in the pyramid) because the algorithm always chooses the static reduction level of the pyramid that is closest to the requested reduction factor. Because the THINNING FACTOR is a constant set at compile time, the computational cost for the dynamic data reduction is also constant—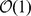.

In summary, both the basic data reduction step and the hierarchical data reduction have constant computational effort; combined, they form the algorithmic framework that permits the panning and zooming of time series data with constant complexity—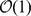.

The initial calculation of the complete data reduction pyramid has a complexity of 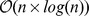, because 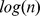 levels need to be computed, each with linear complexity.

### Event data

When working with time series data, events often need to be marked. Events are points in time that can indicate the position of spikes, onsets of stimulation, or other experiment and analysis parameters. In contrast to time series data, event data is sparse; an experiment can contain only a few events (e.g., time points at which the cell culture medium was changed) or a very large number of events (e.g., when marking all spikes). Events are also editable; the user may want to add or remove events during visual inspection.

#### Directory structure

**Figure 4:**
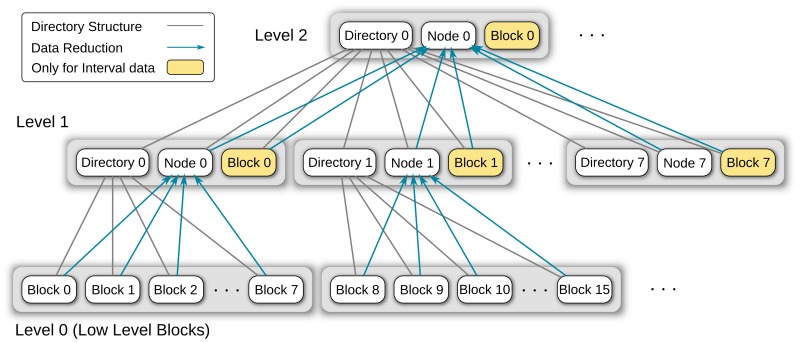
Event and interval data storage structure. Directory structure for the storage of event or interval data. Events or intervals are stored in block files at the bottom at Level 0. Each block file spans a predefined range of samples; all the events or intervals within that range are stored in this block file. The events of all block files in a directory get combined into a node file on the directory level above; data reduction occurs during this step. When storing interval data (yellow boxes), additional block files at higher levels are used to store intervals with end points that do not fit into the sample range of a single block file at the bottom.

**Figure 5:**
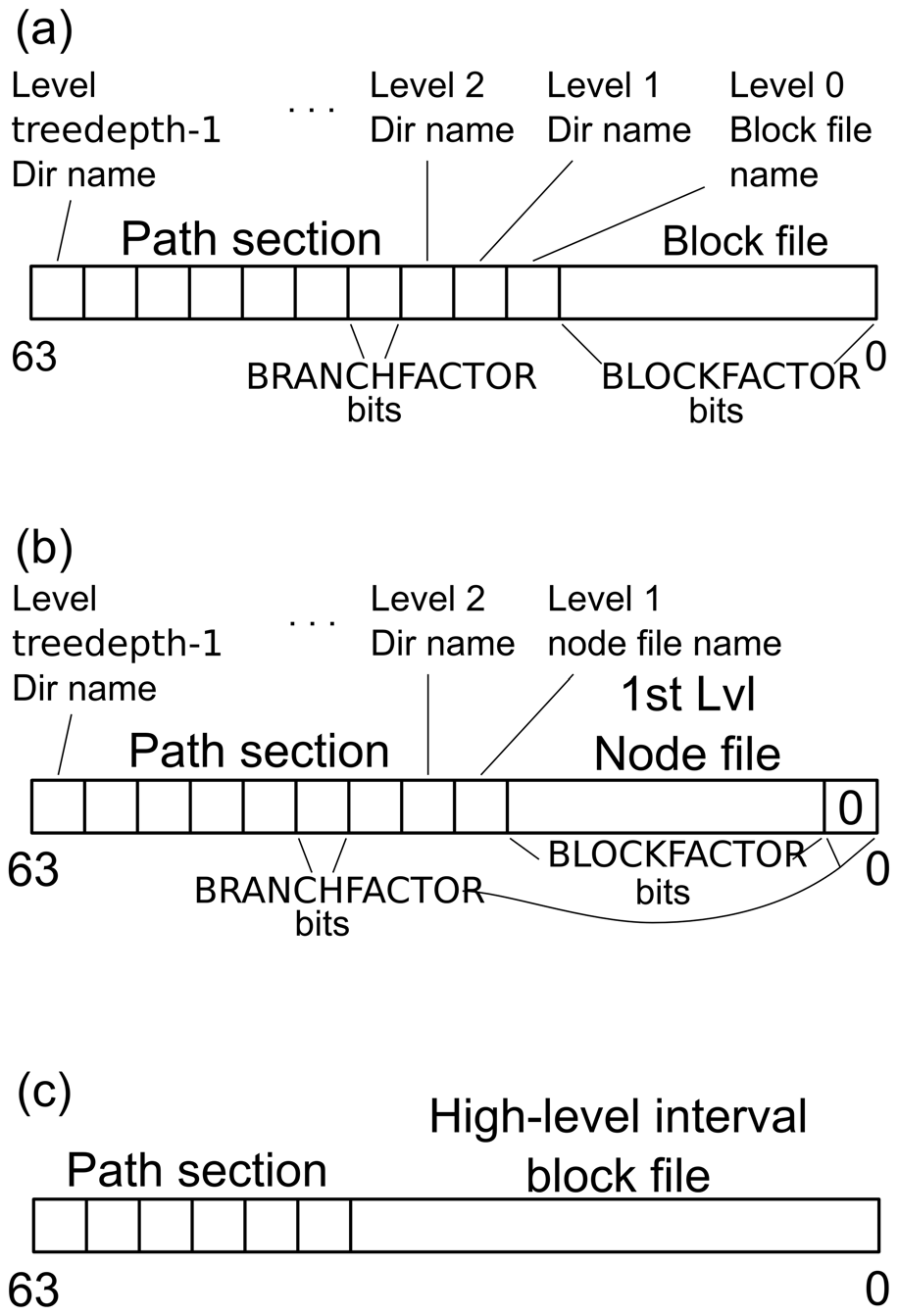
Bit tricks for event and interval data handling. The 64-bit values of events and intervals define the storage location of the value within the directory tree. The high-level bits are divided into chunks of bits with lengths equal to tuning parameter BRANCHFACTOR. When converted to hexadecimal format, they represent the path of the file in which the event or interval is stored. (a) The path of a normal block file comprises treedepth-1 directories and the block file name. Each normal block file spans 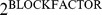 values. (b) Example of a node file on level 1. BRANCHFACTOR low-level bits are set to zero, whereas the path section is shortened by BRANCHFACTOR bits (one directory level). The node files also span 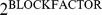 values. (c) [only applies to interval data] If an interval does not begin and end within the same block file on level 0, it gets stored in a high-level block file which offers—depending on its level—a larger value span.

To address these additional requirements, a different storage data structure has been selected. Instead of continuous files, a hierarchical hard disk data storage is used (Fig. 4). Events are stored as 64-bit sample positions in block files on level 0. The storage location of the corresponding block file within the directory tree can be determined from the event value (Fig. 5 (a)). This 64-bit event value is divided into a path section (high-level bits) and a block file section (low-level bits). The path section is divided into chunks with length BRANCHFACTOR, which is a tuning parameter set at compile time. Each chunk is converted into a hexadecimal representation to form a component of the block file path. All events that share the same path and only differ in the block file section are stored in the same block file. The block file section comprises BLOCKFACTOR bits (BLOCKFACTOR is a tuning parameter that is set at compile time); therefore, a *block* file can contain a maximum of 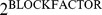 different events.

The fixed association of the event value and storage location within the directory tree facilitates the search of the corresponding block file for the insert and delete operations. The depth of the directory tree is static and calculated from the BLOCKFACTOR and BRANCHFACTOR (see also Fig. 5 (a)):
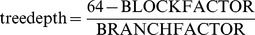
(1)The directory tree is sparse; directories and block files are created on demand when inserting a new event and they are deleted when deleting the last event in a block file or subdirectory.

#### Data reduction step

The visual representation of an event is a vertical line at its (sample) position. Several events projected to the same pixel position on the monitor are indistinguishable from a single plotted event. A large number of events can therefore be replaced by a single vertical line (proxy representation).

**Figure 6:**
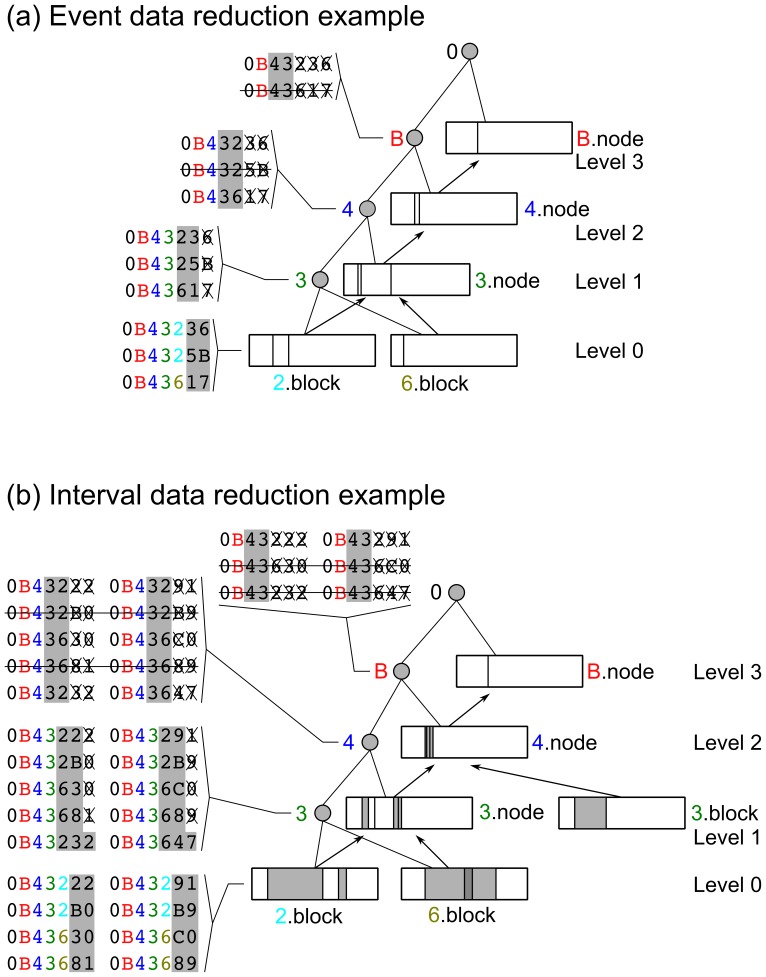
Event data reduction example. (a) shows an example of three events at level 0 and their reduced representations at higher levels of the directory tree. The hexadecimal representation of the events (on the left) correspond to the path to the block file in the directory tree. The gray boxes mark the bits that are encoded within the files and not in the path. Each reduction step from level 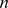 to 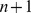 consists of chopping off the last bits (by the amount of BRANCHFACTOR bits; in this example by 4 bits) and then eliminating the duplicate values. (b) Interval data reduction is similar to event data reduction. The large interval (level 0, 2.block, left) gets reduced but still remains an interval on level 1. The smaller interval (level 0, 2.block, right) gets reduced and changes to a single line on level 1 because in the reduced representation, the interval begin and end values collapse to the same value. Overlapping intervals are possible, as shown in level 0, 6.block. The high-level block file 3.block at level 1 contains an interval that would start in 2.block and end in 6.block at level 0. High-level block files, such as 3.block, get combined with the node files at their level (3.node) to form node files at the level above (level 2, 4.node).

The reduced data representations are stored in node files throughout the directory tree and each node file represents its corresponding sub-directory tree (Fig. 4). For the calculation of a node file, all the events from the block and node files in the sub-directory are collected (Fig. 6 (a)). Then, for each event, 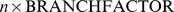 low-level bits are zeroed (see also Fig. 5 (b)); n is the level of the node file in the directory tree. Finally, duplicate events that result from the bit zeroing step are eliminated.

#### Event display list generation

During visualization, the background thread receives requests to prepare new display lists containing the events within a given sample range (defined by the begin and end points) at a certain zoom factor (data reduction level). Owing to the fixed assignment of event position to the block/node file path, the routine can directly calculate the path from where to start the search. Then, it traverses the directory tree at the level corresponding to the requested zoom factor, reading events and proxy representations from the block/node files until the path of the end point is reached. The traversal level is selected so that the static data reduction level of the events and proxy representations is just “below” the requested data reduction level. The events/proxy representations are further reduced (dynamic data reduction) to match the final reduction level (zoom factor) for the visualization and are then added to the display list.

The directory tree can store a maximum of one event at each sample position; therefore, it has the same data density and topology as a time series dataset (compare Fig. 3 and 4). Analog to time series visualization, the combination of basic data reduction step (using a proxy representation to replace several events within one pixel column) and the data reduction hierarchy permits the generation of display lists with constant complexity; panning and zooming of event annotations is possible with constant computational effort—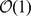.

The generation of the directory tree from a flat file of events takes 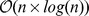 computation time.

### Interval data

Besides marking single time points (events), time series analysis often also requires the intervals of the recordings to be marked, for example, spikes/bursts from their onset to their end, artifacts due to the manipulation of the experiment setup, or interesting periods of the recording. In FTSPlot, intervals are defined by the sample positions of the beginning and end of the intervals. Visually, intervals are represented by semi-transparent colored rectangles that cover the interval range.

#### Directory structure

Interval data is stored in a directory tree structure similar to the directory tree structure for event data (Fig. 4). Intervals are stored as two 64-bit values (begin and end) in block files, with 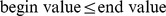. Intervals can overlap.

Special care needs to be taken for intervals that are too large to fit into the range of a single level-0 block file. Such intervals are stored in high-level block files that can be instantiated on demand at each node in the directory tree (Fig. 5 (c)). To determine the corresponding high-level block file for an interval, the common binary prefix of the begin and end values of the interval is computed. The prefix is trimmed to a length equal to a multiple of BRANCHFACTOR bits by cutting away possible surplus low-significant bits. The trimmed prefix is then converted into a directory tree path that points to the node at which the corresponding high-level block file for the interval is located.

#### Data reduction step

Projecting several intervals into the same pixel column is indistinguishable from plotting only a single vertical line into the pixel column. Therefore, intervals are represented by a single vertical line as proxy representation.

The reduction process is analog to event data reduction, except that instead of one 64-bit value for an event, two 64-bit values are processed for an interval. Examples for the reduction process can be seen in Fig. 6 (b). Once the begin and end values of an interval collapse into the same value, the interval is represented by a single proxy line. The additional high-level block files are treated analogously to normal block files for the creation of node files on the level above.

#### Interval display list generation

Interval data display list generation is similar to event data display list generation. During the traversal of block and node files, all intervals are included in the display list that reside either entirely or partially in the requested section.

To include the long-range intervals stored in the high-level block files, a stack is maintained during directory traversal, which contains all the intervals from high-level block files along the path from the root node to the current block/node file. All the long-range intervals intersecting with the requested section are added to form the final display list.

Because the data structure permits overlapping intervals, a block file spanning 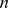 bits can theoretically store a maximum of 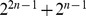 intervals (both the begin and end points can take on all 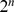 values under the condition that 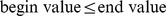). Owing to the large span of the high-level block files and the fact that their intervals are stored on a stack during display list generation, the worst case computational and memory complexity is exponential. However, during normal electrophysiology analysis, such a maximally overlapped interval structure is not to be expected. Rather, it can be assumed that the intervals are mostly non-overlapping and sparse. In this case, the data density and topology resemble time series and event data, and the computational complexity for panning and zooming can be assumed to be constant—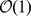.

Under the same assumption of non-overlapping and sparse intervals, the generation of the directory tree from a flat file of intervals takes 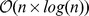 computation time.

### Performance tuning parameters

To achieve quick directory tree traversal during display list generation, the tree structure parameters can be tuned to match the performance characteristics of the used data storage device and file system.

#### • Block file sample range (BLOCKFACTOR)

Controls the sample range covered by the block and node files. An advantage of small ranges is that inserting and deleting events/intervals in small files is quick; however for visualization, numerous small files need to be traversed to create a new display list. This results in more time-intensive directory and inode lookups, leading to poor visualization performance.

Block files that cover a large range on the other side have the advantage of quick visualization; however, they are slow when inserting or deleting events/intervals.

#### • Branch width (BRANCHFACTOR)

Controls the branching width of the directories. Small branch widths result in a deep directory tree that has only a few sub-directories at each node. Large branch widths result in a shallow directory tree and that has many sub-directories at each node.

The optimal value depends on the directory lookup characteristics of the used file system. For file systems that can quickly lookup large directories, a large value is better; however, for file systems that are better at sequentially looking up several smaller directories, a small value performs better.

### Hiding hard disk latency by double buffering

By using the methods described above, the amount of data that is needed to plot a single image is significantly reduced. These small chunks of data can now be loaded on-demand from the hard disk into the main and graphic card memory. To prevent the access latencies of the hard disk from stalling real-time navigation, a display list double buffering system is used (Fig. 1 (b)). While the main thread presents one display list to the user, a background thread fills another display list with new data from the hard disk. The display lists contain more data than required for plotting the current screen section, that is, the display lists are wider and have more resolution than the visible section on the monitor. The extra data acts as a reserve, which permits the user to continue navigating with the old display list, while the background thread prepares the new display list. As long as the new display list is prepared in time, that is, before the user reaches the border of the current display list, the navigation appears to be seamless without a noticeable display list switch. The current implementation works with display lists that are 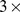 the width and 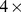 the resolution of the current screen section. Therefore, the display list size depends on the screen width; each display list can contain up to 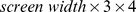 elements (lines/rectangles).

### Implementation

The software was implemented in C++ using the Qt 4.7.0 toolkit for platform independence; a 64-bit operating system is required for memory mapping files 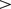
2 GiB. FTSPlot was built, tested, and benchmarked on Linux (gcc 4.4.5); it also successfully built and passed basic tests on Windows 7 (mingw64 or Microsoft Visual Studio 2010) and Mac OS X (Xcode 3); however, thorough tests and benchmarks were not conducted on these platforms.

#### Standalone programs

**FTSPlot**: FTSPlot is the main program and the interactive data viewer. It can display multiple time series, event, and interval datasets in a single graph window and provides intuitive navigation (panning and zooming) using the mouse. For each dataset, it is possible to select the plot color, temporarily exclude the dataset from plotting, and set the plot priority (datasets of high priority are plotted in front of lower priority datasets). The viewer can insert and delete events and intervals and traverse event and interval lists while optionally tracking the current event/interval in the graph window.

**FTSPrep**: Before the time series datasets can be displayed in FTSPlot, they need to be preprocessed to generate the reduced data representations. Depending on the amount of data and hard disk speed, this process can be quite time consuming. Therefore, a separate program—FTSPrep—has been developed for processing time series datasets in batch mode.

#### Application programming interface (API) and Qt widget

To facilitate the adoption of the FTSPlot visualization method, it is possible to embed FTSPlot as a graphical user interface component (Qt GUI widget) into other software projects. In listing 1, a basic example of how to use the API and library is shown.

#include <QApplication>

// Include the header file for the FTSPlot Qt widget

#include <FTSPlot.h>

// all definitions reside in the FTSPlot namespace

using namespace FTSPlot;

int main(int argc, char** argv)

{

// Qt basic setup

QApplication qapp(argc, argv);

// create and show the FTSPlot viewer widget

SimpleViewWidget viewer(NULL);

viewer.show();

// add a time series dataset

viewer.addTimeSeries(“TestDataSet.cfg”);

// zoom to view sample positions 7000–8000

viewer.setXRange(7000, 8000);

// at this point, the GUI setup is complete

// control goes to Qt for user input processing

qapp.exec();

}

**Listing 1**: An example of how to use the FTSPlot API to display a time series dataset

### Benchmarks

The visualization performance of FTSPlot has been evaluated using benchmarks. FTSPlot was connected to a “remote-control” module, which took control of the view navigation, permitting the sequence of display commands in different tests to be replayed reliably. For the benchmarks, a sequence was used, which visualized in each step 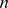 a sample range of length 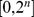 until the entire dataset was covered. In this manner, the sequence permitted the evaluation and comparison of the visualization performance at different magnification levels. The computation time was measured with clock_gettime() for both the preparation of the OpenGL display lists and for the actual painting of the display list. Several factors that can influence the visualization performance have been examined and these factors are listed as follows:

- Tuning parameters

The visualization modules for time series, event, and interval data each have their performance tuning parameters (THINNING FACTOR for time series data, BLOCKFACTOR and BRANCHFACTOR for event and interval data). To determine the optimal values for the tuning parameters, several candidate values have been benchmarked and compared.

- File system cache

Most modern operating systems use the free main memory to cache file system data. This speeds up further read access from the cached data because the main memory is much faster than raw hard disk access. FTSPlot benefits from the file system cache because during normal navigation (panning and zooming), most data is already stored in the file system cache and only small portions of new data need to be read from the hard disk. To estimate the impact of the file system cache, benchmarks have been conducted both with the file system cache and under a setting in which the file system cache is pruned before each test to simulate the “cold-start” conditions. This was achieved by writing a “3” into /proc/sys/vm/drop_caches. Note that this did not prune the read cache of the hard disk itself; therefore, the data cached there might still bias the results.

- Directory and inode block fragmentation

Event and interval display list generation depends on quick directory traversal, which is predominantly influenced by seek times between directory and inode blocks. Fragmented directories require more and longer seeks, increasing the total access time. To estimate the impact of directory and inode fragmentation, benchmarks were conducted with “fragmented” and “optimized” directories.

For generating a fragmented directory, the original directory tree was scanned to obtain a list of all the files and sub-directories. After randomizing this list, the original directory tree was copied to the new directory tree in the randomized order of the list. This ensured—on the tested XFS file system—that the directory and inode blocks in each directory were non-continuous.

For generating optimized directories, the original directory was copied to the new directory in a “depth first search” tree traversal order to achieve a mostly continuous directory and inode block distribution.

The benchmarks were conducted on synthetic datasets. For time series data, a synthetic dataset with a size of 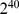 bytes (1 TiB corresponding to 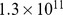 double values) was used and it comprised alternating values of 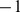 and 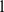.

For event and interval data, the synthetic datasets were designed to be densely filled with events/intervals to simulate the “worst-case performance.” The event dataset contained events at every possible position (sample positions 0, 1, 2, 3,…).

The interval dataset comprised overlapping intervals with variable lengths generated by the formula 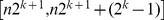, with 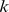 ranging from 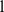 to 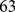 and 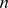 ranging from 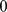 to 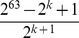. The formula generates interval-gap stipple patterns (50% interval, 50% gap). Parameter 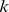 controls the length of the intervals and gaps (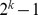). For a given 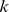, parameter 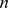 iterates through the individual intervals of the pattern.

Furthermore, for event and interval data, the directory tree was selectively pruned. The benchmark sequence only considers first samples in great detail; samples towards the end of the dataset are only displayed in a “zoomed-out” manner and with significant data reduction. Because the samples towards the end of the dataset are never displayed in great detail, their detailed representations can be omitted to save disk space. In this manner, the benchmark datasets for event and interval data could virtually extend up to 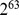 samples.

For evaluating the storage overhead of event/interval directory structures, additional test datasets have been created. They were designed to mimic the analysis situation of a recording from a neuron firing a spike train continuously for 14 days at 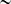
33 Hz frequency. Therefore, the test dataset for event data contained events (detected spikes) every 300 samples (10 kHz sample frequency) and the test dataset for interval data contained intervals of the length of 30 samples every 300 samples.

The storage overhead of time series datasets was determined by measuring the file sizes with the command “ls -l.” For measuring the storage overhead of event/interval directories, the command “df -B 1” was used to determine the amount of used bytes on the hard disk before and after creating the directory tree; the final disk consumption was then calculated by subtracting the two values.

For comparing the FTSPlot time series display to common canonical plotting solutions (gnuplot, matlab, and octave + octplot), benchmarks were conducted with the alternative solutions using increasing sample ranges analog to the FTSPlot benchmarks. The canonical solutions load the complete dataset into the main memory before visualization; therefore, the capacity of the main memory limited the amount of data that could be benchmarked.

All the benchmarks were run in a window with a resolution of 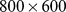 pixels and on a freshly created file system to avoid bias due to unintentional fragmentation. Each benchmark was repeated several times and the results were averaged to even out external influences, such as task-scheduling, interrupts, etc. The number of repetitions was chosen depending on the duration of the specific benchmark; short benchmarks were repeated more often than long-running benchmarks, which automatically evened out sporadic external influences due to their long duration. Time series and cached event/interval benchmarks were repeated 1,000 times, matlab and octave + octplot benchmarks were repeated 100 times and non-cached event/interval and gnuplot benchmarks were repeated 10 times.

- Gnuplot

The Gnuplot benchmarks were controlled by a perl script stepping through the sample ranges and by measuring the elapsed time with the Time:: HiRes:: gettimeofday function. Gnuplot was reading from binary files for improved performance (parsing text files costs additional processing time).

- Matlab

Matlab offers several different plot modes: Hardware OpenGL, Software OpenGL, and zBuffer plotting. For the comparison, Hardware OpenGL was used, which offered the best performance; the results of the other matlab plot modes are not shown. The elapsed time was measured with the tic()-toc() mechanism.

- Octave + octplot

The combination of octave and octplot has been included because it is a free open source solution that uses fast OpenGL plotting. The elapsed time has been measured with the tic()-toc() mechanism.

The specifications of the benchmark test machine are as follows:

A personal computer (PC) with an Intel Core 2 Quad CPU Q9550 CPU @ 2.83GHz, 8 GiB memory, an ATI Radeon HD 4870 graphics card, running Linux with kernel 2.6, and proprietary ATI graphics driver. The data files were stored on a Western Digital WD20EARS hard drive (2 TB, 64 MB cache) using the XFS file system.

FTSPlot has been compiled with gcc 4.4.5 and optimization option -O3. Other software package versions were gnuplot 4.2.6, matlab 2009b, and octave 3.2.3 with an adapted octplot extension.

## Results

A functional prototype was implemented and tested on the 64-bit Linux, Windows, and Mac OS X platforms. Benchmarks were conducted only on Linux.

**Figure 7:**
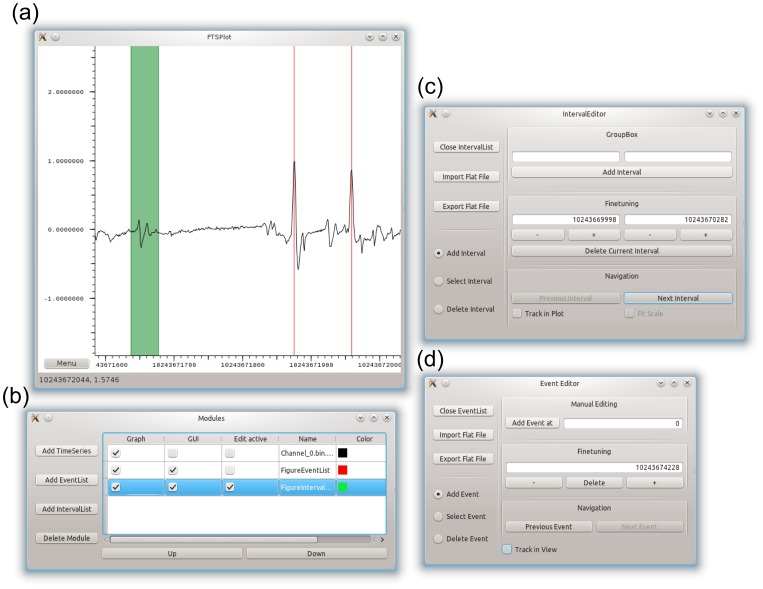
FTSPlot UI screenshots. Screenshots of the FTSPlot user interface during the display of time series, event, and interval data. The main FTSPlot window (a) contains the displayed data and receives mouse and keyboard input for navigation and event/interval editing. In the “Modules” window (b), each data source is represented by a module. For each module, various options are available: toggling its display in the main window (Graph), changing the display color (Color), redirecting the mouse and keyboard input in the main window to a specific event/interval module for editing (Edit active), or opening/closing the option window for a specific event/interval module (GUI). These option windows (c), (d) contain functions for managing the datasets (top left section), selecting/manipulating data in the main window (lower left section), numeric data entry, manipulation, deletion (top right section), and functions to conveniently navigate event/interval lists (lower-right section).

**Figure 8:**
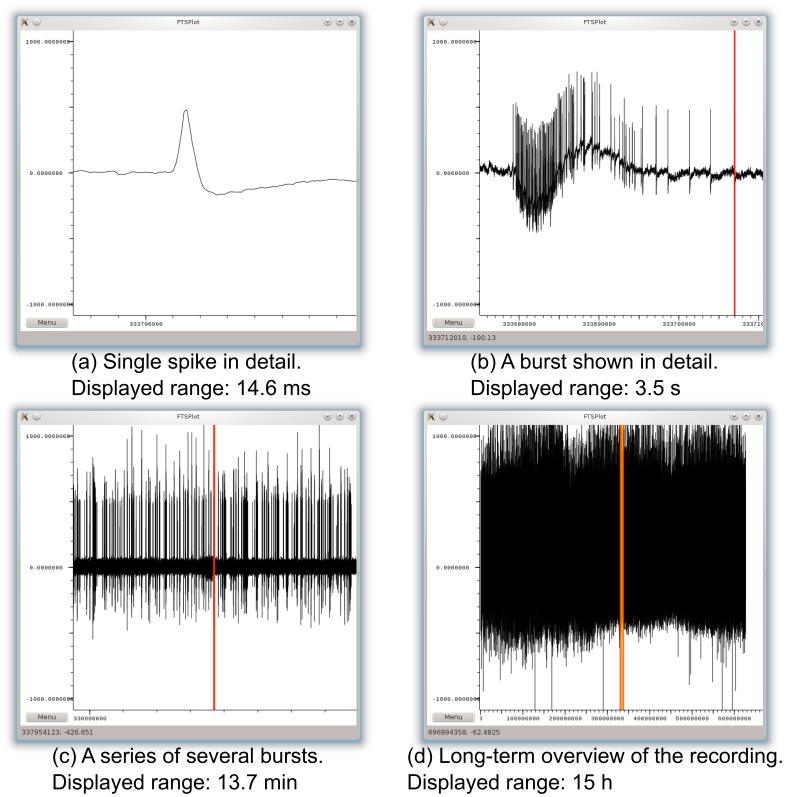
Screenshots with example dataset. Example screenshots of the FTSPlot prototype on Linux showing a real dataset (15 h recording of hippocampal mice neurons in a device with embedded micro-channels [18]). The units on the x-axis are samples; the units on the y-axis are micro-volts. Each of the marked intervals shows the view range of the previous sub-figure. (d) Slight variations of maximum signal amplitude in long-term recordings allow orientation within the data even when the low amplitude signals are too dense and blur together.

Fig. 7 shows the complete user interface. Fig. 8 shows the program displaying an example of a 15 h recording of embryonic mouse hippocampal neurons cultured in a substrate embedded 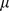-channel device [18]. At each level of magnification, the data viewer offers a natural, artifact-free visual impression of the recording.

### Benchmark results

**Figure 9:**
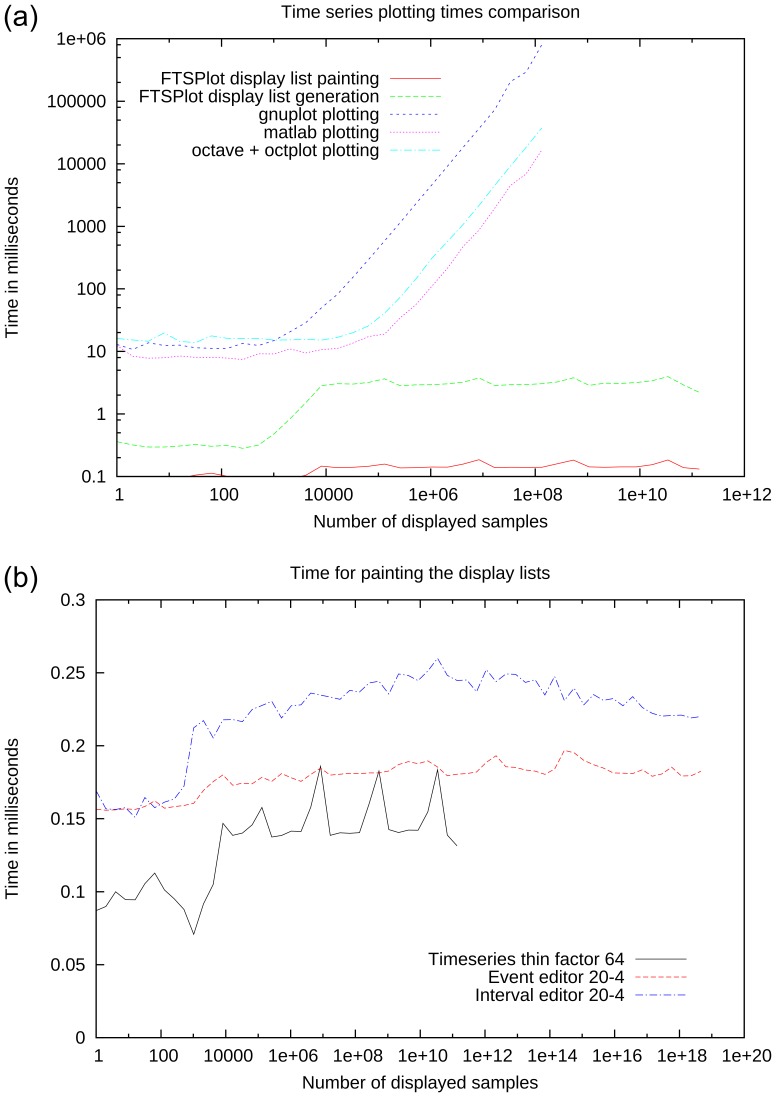
Performance comparison. (a) FTSPlot time series plotting performance (THINNING FACTOR 64) is compared with canonical plotting solutions. Plotting times rise for canonical plotting solutions the more data is displayed, whereas FTSPlot can keep the plotting and display list generation time below 10 ms—independent of the amount of displayed data. (b) shows the time required to paint the display lists for time series, event, and interval data using optimal parameters; overall, they remain below 0.3 ms.

**Figure 10:**
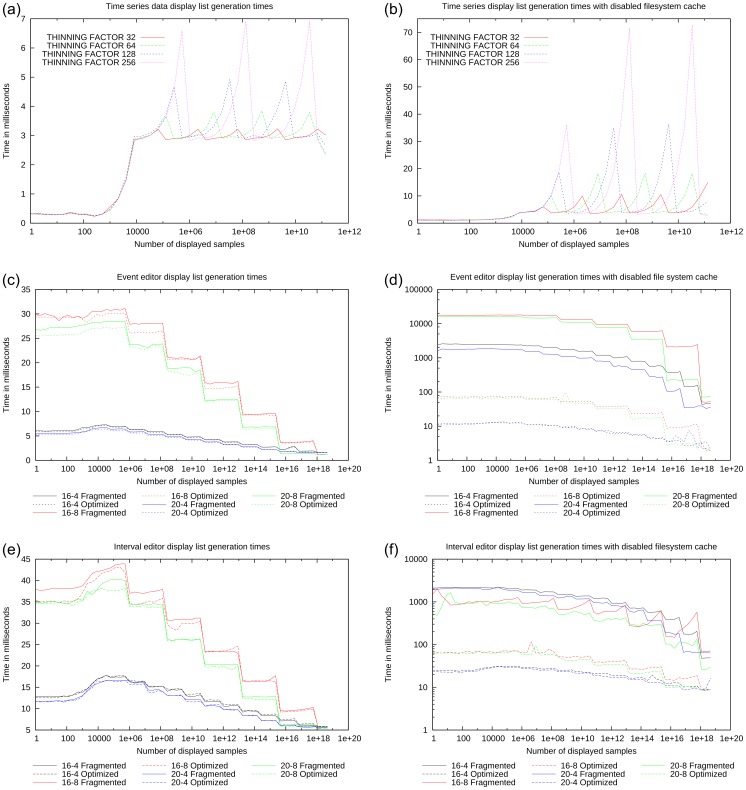
Display list generation benchmark results. Results of the OpenGL display list generation benchmarks for time series, event and interval data, showing the influence of tuning parameters, file system cache, and file system fragmentation. See main text for detailed discussion.

The benchmark results are shown in Figs. 9 and 10. In each figure, the x-axis corresponds to the amount of data that is displayed and the y-axis corresponds to the average processing time required to either plot one image or to generate one display list.

Fig. 9 (a) shows a comparison of the time series plotting performance of FTSPlot and canonical solutions. Overall, the FTSPlot display list generation and painting times remain with 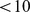 ms below the plotting times of the canonical solutions. For large amounts of data, the plotting time difference is increasing, because for the canonical solutions the plotting times rise with the amount of data, while FTSPlot can cut off the linear rise of display list generation and paint times. The start of the FTSPlot data reduction can be seen to set in at 8,192 samples.

Figs. 9 (b) and 10 show the influence of various parameters on the performance of FTSPlot. In all the graphs, three performance phases can be seen. The first phase for displaying very small amounts of data shows little correlation between the amount of data and display list generation time (e.g., Fig. 10 (a), 0–250 samples). Here, because the amount of processed data is very small, the processing time is dominated by the static function call overhead and therefore remains constant.

In the second phase, the processing time is linearly correlated with the amount of displayed data (e.g., Fig. 10 (a), 250–8,192 samples). In this phase, the amount of data is still small; therefore, the data reduction is not yet active and the processing time shows the typical linear correlation with the amount of data analog to canonical plotting solutions.

In the third phase (e.g., Fig. 10 (a), 8,192+ samples), the data reduction is in effect, the processing time is not correlated to the amount of data anymore, and artifacts that depend on the properties of the data reduction algorithm and underlying data structure are visible.

Fig. 9 (b) shows the OpenGL painting times for time series, event, and interval data. Optimal tuning parameters have been used for each data type (see below). The three phases—constant beginning, linear rise, and plateau—are visible; the overall painting time remains below 0.3 ms.

Fig. 10 contains a grid of benchmark results of OpenGL display list generation performance and how it is affected by various tuning parameters and external conditions. The different data types are arranged top down: times series data (Fig. 10 (a) and 10 (b)), event data (Fig. 10 (c) and 10 (d)), and interval data (Fig. 10 (e) and 10 (f)). The results in the left column (Fig. 10 (a), 10 (c), and 10 (e)) were measured with the activated file system cache, which is the normal condition. The results in the right column (Fig. 10 (b), 10 (d), and 10 (f)) were measured with the deactivated file system cache, simulating the “cold-start” behavior. In each sub-figure, the results for different tuning parameter values are shown; the event and interval data benchmarks additionally distinguish between results from fragmented and optimized file systems.

The detailed results are as follows:

The OpenGL display list generation performance for time series data (Fig. 10 (a), 10 (b)) shows the typical three-phase structure. From 0 to 256 samples, the display list generation time is constant, from 256 to 8,192 samples, no data reduction occurs and the display list generation time is linearly correlated with the amount of data. Data reduction starts at 8,192 samples and the processing time then shows a characteristic saw-tooth profile, which results from the algorithm switching through several levels of statically reduced datasets. In the rising phases, the dynamic data reduction works with the same static reduction level and has to process more data as more samples are displayed, resulting in rising processing time. When switching from one static reduction level to the next, the processing time drops again. Setting the tuning parameter THINNING FACTOR to low values limits the processing time for display list generation but increases the number of reduced datasets (in this example, 5 saw-teeth with THINNING FACTOR  = 32) and the extra storage space (Table 1). Setting the tuning parameter THINNING FACTOR to high values increases the display list generation time but reduces the number of reduced datasets (here, 3 saw-teeth with THINNING FACTOR  = 256) and extra storage space.

**Table 1:** Time series data overhead.

Thinning factor	Relative overhead
32	6.5%
64	3.2%
128	1.6%
256	0.8%

When conducting the benchmarks with the deactivated file system cache, the display list generation time rises in general (Fig. 10 (b)). Moreover, the first saw-tooth is now smaller than the following ones. This is because the first dynamic data reduction phase uses the original dataset with one 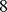-byte floating point value per sample compared to 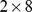 bytes floating point values per sample in the reduced (min/max) datasets for the following dynamic data reduction phases. Because the display list generation speed without the file system cache depends mainly on the reading speed of the hard disk, this difference is reflected in the display list generation times.

Figs. 10 (c), 10 (d), 10 (e), and 10 (f) show the OpenGL display list generation times for event and interval data. The third phase (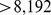 samples) shows a characteristic stepwise decrease in processing time. This is because the node files with reduced data are stored closer to the root node than the block files. Therefore, when displaying more data, the directory traversal from the tree root to the node files becomes shorter and the display list generation time becomes stepwise faster whenever the traversal switches to the next higher directory level. With the file system cache, the fragmentation level of the directory and inode blocks bears only a slight influence on the processing times, which shows that the data is predominantly served from the file system cache (Fig. 10 (c), 10 (e)). Without the file system cache, directory fragmentation bears a strong influence on processing time, because data is directly read from the hard disk and access times depend on the directory and inode block layout. Optimized directory and inode block layouts can be read faster than fragmented block layouts.

### Storage overhead

The statically reduced datasets (proxies) that are required for accelerating visualization are stored on the hard disk and consume extra space. Table 1 shows the relative storage overhead for time series data depending on the used THINNING FACTOR. Determining the storage overhead for event and interval data is more difficult because storage requirements for directories largely depend on the used file system and how it organizes its internal data structures. Moreover, events and intervals are sparsely distributed, which leads to

- small files, which are often too small to fill up a complete physical block on the hard disk, resulting in poor disk utilization and higher disk space consumption.
- a multiplication of data; from each block file, a chain of node files extends towards the root of the directory tree, consuming additional disk space.

Two types of datasets were used to evaluate the storage overhead: “regular” datasets, which were used for simulating a spike train analysis (see methods section), and the “sparse” pruned directory datasets, which were used for the benchmarks. The results are shown in Table 2. Although the regular datasets showed a noticeable storage overhead of up to four times the original dataset size, the sparse datasets drastically raised the disk space consumption up to 2,175 times the original dataset size. Here, the artificially sparse nature of the pruned directory tree leads to very small and distributed file and directory entries. Together with a relatively large disk block size of 4,096 bytes (compared to 8 or 16 bytes for a single event/interval entry), this results in a huge storage overhead.

**Table 2:** Event and interval data overhead.

Type, block/branch parameters	Regular dataset overhead	Sparse dataset overhead
Event, 16–4	378%	217481%
Event, 16–8	253%	118621%
Event, 20–4	212%	176257%
Event, 20–8	117%	96915%
Interval, 16–4	253%	8696%
Interval, 16–8	128%	4032%
Interval, 20–4	213%	8064%
Interval, 20–8	104%	3780%

In a more realistic, non-sparse dataset, events/intervals can group more closely together and start sharing the same resources (using the same disk blocks if they are in the same block file, or sharing the same directory and node file blocks when they are in the same sub directory tree).

### Optimal tuning parameters

From the benchmark results, optimal parameters can be determined for the fine-tuning parameters.

For time series data, 64 was chosen as the optimal value for tuning parameter THINNING FACTOR. It is the best compromise between speed (paint time 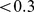 ms; OpenGL display list generation time 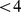 ms with the file system cache and 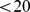 ms without the file system cache) and storage overhead (3.2%).

For event and interval data, the storage overhead did not influence the decision of the optimal tuning parameters because the amount of event and interval data is assumed to be small and therefore negligible compared to the storage requirements for the time series data. Therefore, only visualization speed and event/interval insertion/deletion speed (results not shown) were considered for selecting the optimal tuning parameters, resulting in BLOCKFACTOR  = 20 (max. 1,048,576 samples) and BRANCHFACTOR  = 4 (max. 16 directories) for both event and interval data.

## Discussion

The FTSPlot project has shown that by using preprocessed datasets the exploration of time series data, event and interval annotations is possible in constant time—
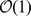
—and is therefore independent of the amount of data. Display list generation times of 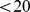 ms and painting times of 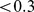 ms support fast and continuous interactive navigation of long-term electrophysiological recordings with at least 50 frames per second (excluding cold-start conditions). The high drawing speed ensures visual object constancy and avoids change blindness effects. The necessary preprocessing step to prepare the datasets for visualization has a complexity of 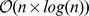 and can be conducted unattended in a batch process.

As compared to canonical plotting solutions, which slow down as more data is plotted, this represents a qualitative improvement of the algorithmic complexity during visualization from 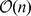 to 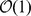.

In practice, this improvement becomes noticeable when working with datasets beyond 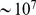 samples (e.g., recordings 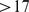 minutes at 10 kHz) because then the plotting time with canonical solutions rises above 1 second and starts hindering continuous, interactive data navigation (Fig. 9 (a)).

### Limitations

#### x86_64 48-bit virtual addresses

In theory, using a 64-bit architecture should allow FTSPlot to map datasets with up to 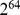 bytes into the virtual address space. In practice, current implementations of the x86_64 architecture often only use 48 bits for virtual addresses[39]. This limitation reduces the amount of data that can be addressed from 16 EiB (64 bit) to 256 TiB (48 bit), which corresponds to 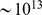 double values. In reality, this amount will be even lower because the x86_64 address space is split into two halves and the address ranges for the operating system and program code occupy additional address space. The benchmark above was conducted with 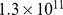 double values occupying 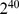 bytes, which is already close to the practical 48-bit limit. Considering the continuously growing storage capacities, newer processors in the future are expected to have an extended virtual address space beyond 48 bits. An alternative to expanded address ranges would be to change the program to use seek/read semantics instead of memory mapping.

#### Visibility

The objective of the FTSPlot project was to accelerate time series plotting, while still maintaining the visual result of a canonical line plot. The practical use of FTSPlot with extra-cellular electrophysiology recordings has shown that at each zoom level, it is possible to present sensible information regarding the features at that aggregation level: spike shapes, spike bursts, and the overall development of spike amplitudes (Fig. 8). In general, however, plotting large amounts of data can lead to a crucial loss of information due to overdraw. In particular, periodic signals with large amplitudes (e.g., artifacts of regular external stimulation) suffer from a banding effect that inhibits sensible visualization when plotting data at overview zoom levels. Further research is required to determine the right visual cues (color gradients, intensity, transparency, stipple patterns, and multi-plots with mean, min, max, and standard deviation values [24], [29]) to express sensible information in such situations. For very large datasets, the cues are likely to also require information about semantic properties corresponding to the specific type of data and analysis step, for example, average spike amplitudes, spike densities, and number of spikes per spike train.

### Further performance considerations

The computational effort required to prepare the reduced datasets with FTSPrep is very small; therefore, the limiting factor for the static data reduction is the sequential reading speed of the hard disk. For example, preparing a single channel recording of 1 day (6.4 GiB) takes 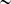
1.3 minutes assuming a hard disk throughput of 85 MiB/sec. In subsequent versions of the software, the data reduction may even be included into the recording and analysis software so that the reduction can occur “on the fly” during recording/analysis.

The storage overhead for reduced time series datasets remains below 4% of the original data size (using the optimal THINNING FACTOR of 64). In contrast, the storage overhead for event and interval datasets cannot be bounded by an upper limit because it depends on the sparseness of the data and on the used file system. In extreme cases, the storage overhead can grow drastically with very sparse data (Table 2); however, for practical applications, the storage overhead should remain below 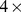 the size of the original dataset. Although considering today's hard disk capacities and prices, this overhead is acceptable in most cases, the increased disk space consumption should be considered when planning experiment and analysis steps that generate considerable event or interval data.

Care needs to be taken to ensure that the data hard disks are used exclusively during visualization. Multi-user/multi-program access to the data hard disk can lead to increased access times that the double buffering strategy of FTSPlot may not be able to compensate. Although time series visualization is quite well adapted to the sequential access characteristics of hard disks, event and interval visualization with its random access pattern may further benefit from Solid State Disks (SSDs) that offer faster random access.

A requirement of the current implementation is the use of a 64-bit operating system for memory mapping large data files. This has the additional advantage that the operating system can provide an optimal caching strategy at the system level. If FTSPlot is exclusively used on the system, it can use the entire available main memory for caching; however, if FTSPlot is idle and other programs are active and filling the file system cache, its effective memory consumption is reduced.

FTSPlot is offered as a free standalone software for evaluating its capabilities and as software component (Qt widget) for inclusion in further projects. The source code is available from https://github.com/MichaelRiss/FTSPlot. Together with high performance data analysis components, FTSPlot can form a software infrastructure for analyzing large-scale time series data. This should permit the continuous monitoring of electrophysiological experiments towards understanding long-term processes of neural growth, plasticity, degeneration, and regeneration.
